# Auditory-motor adaptation: induction of a lateral shift in sound localization after biased immersive virtual reality training

**DOI:** 10.3389/fcogn.2024.1400292

**Published:** 2024-07-31

**Authors:** Alma Guilbert, Tristan-Gael Bara, Tifanie Bouchara

**Affiliations:** ^1^Laboratoire Vision Action Cognition (VAC), Université Paris Cité, Boulogne-Billancourt, France; ^2^Centre d'Études et de Recherche en Informatique et Communications (CEDRIC), Conservatoire National des Arts et Métiers (CNAM), Paris, France; ^3^Laboratoire Interdisciplinaire des Sciences du Numérique (LISN), Université Paris-Saclay, CNRS, Orsay, France

**Keywords:** immersive virtual reality, HRTF, hearing, sound localization, prism adaptation, sensorimotor adaptation

## Abstract

**Introduction:**

Sensorimotor adaptation has often been studied in the visual modality through the Prism Adaptation (PA) paradigm. In this paradigm, a lateral shift in visual pointing was found after wearing prismatic goggles. An effect of PA has sometimes been observed on hearing, in favor of a cross-modality recalibration. However, no study has ever shown if a biased auditory-motor adaptation could induce this lateral shift, which appears essential to a better understanding of the mechanisms of auditory adaptation. The present study aimed at inducing an auditory prism-like effect.

**Methods:**

Sixty healthy young adults underwent a session of active audio-proprioceptive training in immersive virtual reality based on Head Related Transfer Functions (HRTF). This training consisted of a game in which the hand-held controller emitted sounds either at its actual position in a control group or at 10° or 20° to the right of its actual position in two experimental groups. Sound localization was assessed before and after the training.

**Results:**

The difference between both localization tests was significantly different between the three groups. As expected, the difference was significantly leftward for the group with a 20° deviation compared to the control group. However, this effect is due to a significant rightward deviation in the control group whereas no significant difference between localization tests emerged in the two experimental groups, suggesting that other factors such as fatigue may have cumulated with the training after-effect.

**Discussion:**

More studies are needed to determine which angle of deviation and which number of sessions of this audio-proprioceptive training are required to obtain the best after-effect. Although the coupling of hearing and vision in PA still needs to be studied, adding spatial hearing to PA programs could be a promising way to reinforce after-effects and optimize their benefits.

## 1 Introduction

Spatial cognition is mainly studied in the visual modality while it is a multisensory process also based on hearing. Fundamental differences exist between vision and hearing for space, with the visual modality being more reliable in spatial localization and the auditory modality being more temporal-based (Blauert, 1996). Indeed, visual perception always involves spatial processing because projections on the retina are inherently spatial. On the contrary, spatial position in hearing is only computed a posteriori thanks to several auditory cues (binaural and spectral cues). These cues are contained in the Head Related Transfer Function (HRTF) simulating the transformations due to the auricular pinna, head, and torso, for a sound-given position and are, thus, individual.

Obtaining individual HRTF is a complex process and requires time and specific materials like an anechoic chamber. Due to these constraints, non-individual HRTF are often used in Virtual Reality (VR) applications and the use of these unfamiliar HRTF can lead to poor sound localization. However, previous studies have demonstrated that the auditory system can learn and adapt to new HRTF, non-individualized or altered (Parseihian and Katz, 2012; Carlile and Blackman, 2014; Steadman et al., 2017; Stitt et al., 2019). For instance, in Carlile and Blackman's (2014) study, eight participants wore earmolds degrading HRTF and, therefore, impaired sound localization for 28–62 days. Over time, results showed a significant improvement in sound localization with earmolds. The duration of adaptation can be shortened with active and implicit gamified training (Parseihian and Katz, 2012; Steadman et al., 2017; Bouchara et al., 2019; Stitt et al., 2019). The training program mainly consisted of sessions of a 12-min VR version of a hot and cold game where blindfolded participants explore the space around them to search for targets using a position-tracked ball or a controller in their hand (Parseihian and Katz, 2012; Bouchara et al., 2019; Stitt et al., 2019). The distance between the hand and the target is sonified thanks to non-individual HRTF. Carried out in three sessions on 3 consecutive days (Parseihian and Katz, 2012; Bouchara et al., 2019) or up to 10 sessions at intervals of 1 or 2 weeks (Stitt et al., 2019), results showed HRTF adaptation with better sound localization in VR after this training, suggesting a realignment between hearing and proprioception. Thus, the auditory system is plastic and highly adaptable to environmental changes. This adaptation could be even more important than the visual one as sound localization is less reliable, and, thus, could be easier to change (Burge et al., 2010).

Sensorimotor adaptation is already often studied in the visual modality through the prism adaptation (PA) model (Redding et al., 2005; Michel, 2016). The PA paradigm involves an adaptation phase during which the participant wears prismatic goggles that deviate the entire visual field usually 10° to the right in case of rightward PA. The participant engages in a sensorimotor task such as pointing to visual targets while wearing the goggles. After an initial phase in which the participant overshoots the targets to the right, the pointing becomes correct with practice. After the removal of the goggles, the adaptation effect persists, and the participant points to the target with a leftward bias, i.e., in the opposite direction to the prismatic deviation. This after-effect is not permanent but varies in time depending on the number of sessions and time exposure (Schintu et al., 2014). PA is considered a learning process that minimizes disparities between vision and position sense, corresponding to proprioception (Kornheiser, 1976).

Several studies also underlined an effect of PA on other sensory modalities than vision suggesting a cross-modality recalibration (McIntosh et al., 2002; Girardi et al., 2004; Cui et al., 2008). In healthy participants, after-effects of PA were observed on haptic tasks (Girardi et al., 2004), suggesting that PA effects can extend to unexposed sensory systems. However, the effect of PA on hearing was essentially studied in patients who suffered from Unilateral Spatial Neglect (USN; Jacquin-Courtois et al., 2010; Tissieres et al., 2017; Matsuo et al., 2020).

USN is a neuropsychological syndrome affecting spatial cognition and characterized by a failure to respond, orient, or initiate action toward contralesional targets, mainly consecutive to a cerebral stroke (Heilman and Valenstein, 1979). The effects of PA have been extensively studied in USN as PA is commonly used as a rehabilitation program for this population (Pisella et al., 2006; Li et al., 2021). A decrease in visual USN symptoms has been well-documented after PA toward the ipsilesional space (Jacquin-Courtois et al., 2013; Li et al., 2021), suggesting strong links between low-level sensorimotor plasticity and high-level cognitive functions (Jacquin-Courtois et al., 2013; Michel, 2016). In USN, PA not only decreases visual symptoms but also auditory extinction, which corresponds to the failure to hear a contralesional sound when presented simultaneously with an ipsilesional sound (Jacquin-Courtois et al., 2010; Tissieres et al., 2017). However, auditory extinction is not a synonym of USN, which can be characterized by auditory symptoms such as sound localization difficulties (Pavani et al., 2004; Guilbert et al., 2016). While Tissieres et al. (2017) did not find improvement, but rather detrimental effects, of PA on sound localization in USN, Matsuo et al. (2020) found a beneficial effect of PA on sound localization even with a single session. Tissieres et al. (2017) suggested that their absence of results could be due to the complex nature of auditory space encoding at a cortical level. Thus, it is unclear how auditory spatial localization can be laterally shifted by PA. Moreover, while the benefits of auditory-motor adaptation on sound localization have been explored (Parseihian and Katz, 2012; Bouchara et al., 2019; Stitt et al., 2019; Valzolgher et al., 2020), to the best of our knowledge, no study has ever shown whether a biased auditory-motor adaptation could induce a lateral shift of sound localization, which is of major interest to understand the mechanisms of auditory adaptation better and optimize its use in multisensory training.

The present study aimed at inducing a prism-like effect in the auditory modality in healthy young adults. Participants were divided into three groups and went through a short session of active audio-proprioceptive training with non-individual but selected HRTF inspired by previous studies (Parseihian and Katz, 2012; Bouchara et al., 2019; Stitt et al., 2019), in which their hand was sonified either at their actual position in a control group or at 10° or 20° to the right of their actual position in two experimental groups. After training, both experimental groups were expected to show a leftward bias in sound localization compared with initial pre-training performance, albeit with a larger effect in the group with the 20° bias. In contrast, no deviation in sound localization was expected in the control group.

## 2 Materials and methods

### 2.1 Participants

Sixty participants were recruited. All were Psychology students and had no history of neurological or psychiatric illness. They also had normal hearing tested through an audiometric test before the experiment (pure-tone thresholds ≤ 20 dB for frequencies 500, 1,000, 2,000, 4,000, and 8,000 Hz). Participants were randomly assigned to three groups of 20 participants. The three groups differed by the deviation of the sonification induced during the adaptation task. In the control group (G_C_), no deviation was induced. In the 10° deviation group (${\text{G}}_{10^{{}^{\circ}}}$), a deviation of 10° was induced to the right. In the 20° deviation group (${\text{G}}_{20^{{}^{\circ}}}$), a deviation of 20° was induced to the right. There was no significant difference in the mean age between the three groups (G_C_: M = 19.1 years, SD = 1.14; ${\text{G}}_{10^{{}^{\circ}}}$: M = 20.0 years, SD = 2.39; ${\text{G}}_{20^{{}^{\circ}}}$: M = 20.5 years, SD = 1.96). Each group was composed of 16 females and four males. Seventeen participants were right-handed and three were left-handed in G_C_ and ${\text{G}}_{10^{{}^{\circ}}}$ while 19 participants were right-handed, and one was left-handed in ${\text{G}}_{20^{{}^{\circ}}}$.

### 2.2 Materials

Participants carried out two different tasks adapted from previous studies (Parseihian and Katz, 2012; Bouchara et al., 2019; Stitt et al., 2019): one training task for the adaptation to HRTF and one sound localization task to assess this adaptation. The audio-virtual environments were developed under Unity with Steam VR and were rendered using an HTC Vive Pro as a head- and hand-tracker. Sennheiser HD 380 Pro headphones were used to present auditory stimuli. 3D audio spatialization was obtained through the Steam Audio Plugin using SOFA HRTF files from the public LISTEN library of HRTF (Warusfel, 2003). To minimize interindividual differences in HRTF, each participant carried out a perceptive judgment task on seven pairs of HRTF selected in the LISTEN library to select the one they used for the rest of the experiment in both the localization test and the adaptation task (for detailed procedure see Bara et al., 2020).

### 2.3 Procedure

**Figure 1:**
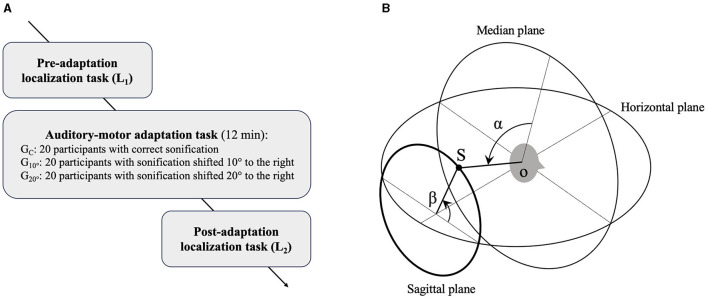
Schematic illustrations of **(A)** experimental procedure and **(B)** polar coordinate system. S, sound source; o, center of the head; α, lateral angle; β, polar angle (inspired by Morimoto and Aokata, 1984).

The procedure is presented in Figure 1A. Participants were tested individually in a quiet room, seated in a swivel chair. They carried out a first localization test (L_1_) to assess their initial performance with the pair of HRTF selected. Then, they went through one session of the adaptation task. Contrary to previous studies that used several sessions (Parseihian and Katz, 2012; Bouchara et al., 2019; Stitt et al., 2019), only one single session was used here. A second localization task (L_2_) to assess sound localization performance was performed 3 min after the end of the adaptation task.

In the localization task, participants had to report the perceived position of a static spatialized sound sample by pointing in its direction with the controller held in their dominant hand and validating with the trigger. The stimulus consisted of a train of three 40 ms Gaussian broadband noise bursts (50–20 000 Hz) separated by 30 ms of silence. Each localization test was composed of two blocks of 33 trials testing localization performance for 11 azimuths (−90°, −72°, −54°, −36°; −18°, 0°, +18°, +36°, +54°, +72°, +90°) and 3 elevations (-30°, 0°, +30°). In each of the two blocks, trials were randomly presented. Each new trial began with the pointing of a target (green sphere) presented visually at a position of 0° azimuth and 0° elevation, so participants were always oriented similarly at the beginning of a trial. No other visual information was displayed during the localization task.

In the adaptation task, participants had to freely scan the surrounding space with their controller held in their dominant hand to find animal sounds hidden randomly in the frontal hemisphere Participants were asked to find as many targets (sounds of animals) as they could for 12 min. The controller-to-target angular distance was sonified through the alternate speed between white and pink noise such as the delay between each burst decreased from 3 s to 0.05 s with the angular distance. When the target was reached, a random animal sound (in a set of 17) was played through the headphones and a new trial began. The feedback and the animal sounds were spatialized through HRTF at the actual controller position for G_c_, 10° to the right of the controller for ${\text{G}}_{10^{{}^{\circ}}}$ or 20° to the right of the controller for ${\text{G}}_{20^{{}^{\circ}}}$. No visual information was displayed in the HTC Vive Pro during the adaptation task.

### 2.4 Analysis of results

Target and response azimuths and elevations were logged during the localization tasks for each trial. These measures were converted into the interaural polar coordinate system (Morimoto and Aokata, 1984; Parseihian and Katz, 2012). In this coordinate system, azimuth and elevation angles are transformed into lateral and polar angles (see Figure 1B). Lateral angles are the angles between the median plane and the line connecting the target with the center of the participant's head and vary between −90° and 90° from left to right. Polar angles correspond to rotation around the interaural axis, from −90° to 270° with 0° in front of the participant. This coordinate system permits a rough separation between binaural disparity cues that determine lateral angles and spectral cues that determine polar angles. All front/back confusion errors, which are frequent in sound localization, are, thus, contained in the polar angles. Localization errors in lateral and polar angles correspond to the difference between the target and perceived angles. Thanks to the interaural polar coordinate system, lateral errors could reveal a lateral shift without any influence of front/back confusions, which is not the case with azimuths. The mean of absolute lateral and polar errors and the percentage of front-back errors (calculated as in Parseihian and Katz, 2012) were first analyzed. To account for within-subject variance while modeling between-subject differences, a mixed linear model (MLM) was carried out with Localization test (L_1_ and L_2_) and Group (G_C_, ${\text{G}}_{10^{{}^{\circ}}}$, and ${\text{G}}_{20^{{}^{\circ}}}$) as the fixed factors and Participant as the random factor on these measures to ensure that the three groups were equivalent. The same MLM was carried out on lateral errors to ensure the presence of an interaction effect between the fixed factors Localization test and Group. Other MLM were also carried out separately for each group with Localization test (L_1_ and L_2_) as the fixed factor and Participant as the random factor to search for potential lateral shifts. Before these analyses, an analysis of variance (ANOVA) was also performed on the number of target sources found during the adaptation task to ensure the equivalence of the three versions (this data was not available for four participants of ${\text{G}}_{10^{{}^{\circ}}}$ due to incomplete logging). All assumptions for carrying parametric analyses were fulfilled. A significance threshold of 0.05 (two-tailed alpha level) was adopted for all analyses.

## 3 Results

For the number of targets found during the adaptation task, no significant difference (*F*_(2, 53)_ = 0.70, *p* = 0.50) was observed between G_C_ (M = 13.2, SD = 6.76), ${\text{G}}_{10^{{}^{\circ}}}$ (M = 12.0, SD = 3.85), and ${\text{G}}_{20^{{}^{\circ}}}$ (M = 11.3, SD = 3.93).

Results for lateral errors, absolute lateral and polar errors, and front-back confusions in the localization tasks are reported in Table 1. Statistical results for fixed effects in the MLM are also reported in Table 1.

**Table 1:** Mean and standard deviations of lateral errors, absolute lateral and polar errors (in °), and front-back confusions (in %) for each Group (G_C_ = control group, ${\text{G}}_{10^{{}^{\circ}}}$ = group with 10° lateral deviation, ${\text{G}}_{20^{{}^{\circ}}}$ = group with 20° lateral deviation) and Localization test (L_1_ = first localization test, L_2_ = second localization test). Mixed linear model (MLM) fixed effects are also reported (^*^p < 0.05, ^**^p < 0.01).

	**G**_**C**_		${\text{G}}_{10^{{}^{\circ}}}$		${\text{G}}_{20^{{}^{\circ}}}$		
	**L**_1_	**L**_2_	**L**_1_	**L**_2_	**L**_1_	**L**_2_	**MLM fixed effects**
Lateral errors (°)	1.76 (±6.69)	5.72 (±6.91)	1.87 (±8.27)	2.83 (±6.97)	−0.74 (±4.79)	−2.36 (±5.90)	Group: *F*_(2, 57)_ = 3.96, *p* = 0.024^*^, $\eta_{\text{p}}^{2}$ = 0.12
							Localization test: *F*_(1, 57)_ = 3.19, *p* =0.080
							Group × Localization test: *F*_(2, 57)_ = 6.81, *p* = 0.002^**^, $\eta_{\text{p}}^{2}$ = 0.19
Absolute lateral errors (°)	22.4 (±4.50)	22.5 (±6.25)	19.8 (±4.53)	20.5 (±6.09)	18.6 (±4.90)	17.1 (±4.62)	Group: *F*_(2, 57)_ = 4.70, *p* = 0.013^*^, $\eta_{\text{p}}^{2}$ = 0.14
							Localization test: *F*_(1, 57)_ = 0.20, *p* = 0.66
							Group × Localization test: *F*_(2, 57)_ = 1.77, *p* = 0.18
Absolute polar errors (°)	109.7 (±28.9)	106.9 (±30.4)	91.7 (±35.6)	91.8 (±37.4)	115.9 (±35.9)	112.6 (±39.0)	Group: *F*_(2, 57)_ = 2.38, *p* = 0.10
							Localization test: *F*_(1, 57)_ = 0.89, *p* = 0.35
							Group × Localization test: *F*_(2, 57)_ = 0.25, *p* = 0.78
Front-back confusions (%)	36.1 (±19.4)	32.3 (±17.5)	26.0 (±21.8)	23.7 (±21.4)	26.8 (±19.0)	25.7 (±20.9)	Group: *F*_(2, 57)_ = 0.28, *p* = 0.28
							Localization test: *F*_(1, 57)_ = 3.69, *p* = 0.060
							Group × Localization test: *F*_(2, 57)_ = 0.38, *p* = 0.69

No significant effect emerged from the MLM for absolute polar errors and front-back confusions (see Table 1). These measures did not significantly differ between groups or localization tests. For absolute lateral errors, only a significant main effect of the factor Group emerged (see Table 1). The absolute lateral errors were significantly smaller in ${\text{G}}_{20^{{}^{\circ}}}$ than in G_C_ (*t* = −3.05, *p* = 0.003). ${\text{G}}_{10^{{}^{\circ}}}$ did not significantly differ from G_C_ (*t* = −1.80, *p* = 0.077) or ${\text{G}}_{20^{{}^{\circ}}}$ (*t* = 1.25, *p* = 0.22).

The results of the MLM on lateral errors are displayed in Table 2. The MLM analysis did not yield a main effect of the fixed factor Localization test. However, a significant main effect of the fixed factor Group emerged (see Table 1). The model revealed that the lateral errors were significantly more to the left in ${\text{G}}_{20^{{}^{\circ}}}$ than in G_C_ (*t* = −2.72, *p* = 0.009) and in ${\text{G}}_{10^{{}^{\circ}}}$ (*t* = 2.00, *p* = 0.050). ${\text{G}}_{10^{{}^{\circ}}}$ did not significantly differ from G_C_ (*t* = −0.72, *p* = 0.48). This main effect of the factor Group was explained by a significant Group × Localization test interaction in the model (see Table 2).

**Table 2:** Fixed effects for linear mixed model predicting lateral errors (^**^p < 0.01, ^***^p < 0.001).

**Parameter**	**Estimate**	**Standard error**	**95% confidence interval**	***t***	***p***
Intercept	1.51	0.80	[−0.05, 3.07]	1.90	0.062
**Group**					
${\text{G}}_{10^{{}^{\circ}}}$-G_C_	−1.39	1.95	[−5.21, 2.42]	−0.72	0.48
${\text{G}}_{20^{{}^{\circ}}}$-G_C_	−5.29	1.95	[−9.11, −1.47]	−2.72	0.009^**^
**Localization test**					
L_2_-L_1_	1.10	0.62	[−0.11, 2.31]	1.79	0.080
**Group** × **Localization test interaction**					
${\text{G}}_{10^{{}^{\circ}}}$-G_C_ × L_2_-L_1_	−3.00	1.51	[−5.96, −0.03]	−1.98	0.053
${\text{G}}_{20^{{}^{\circ}}}$-G_C_ × L_2_-L_1_	−5.58	1.51	[−8.55, −2.61]	−3.69	< 0.001^***^

**Figure 2:**
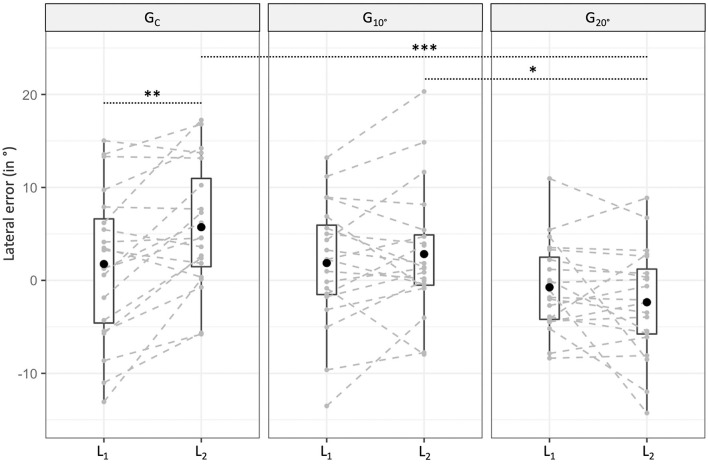
Box plot of lateral errors for each group (G_C_ = control group, ${\text{G}}_{10^{{}^{\circ}}}$ = group with 10° lateral deviation, ${\text{G}}_{20^{{}^{\circ}}}$ = group with 20° lateral deviation) and localization test (L_1_ = first localization test, L_2_ = second localization test). The dark point denotes the average value, while the lower and upper hinges correspond to the first and third quartiles. The upper whisker extends from the hinge to the largest value and the lower one to the smallest value (within the 1.5 interquartile range from the hinge). Individual results are displayed in gray (*p < 0.05, **p < 0.01, ***p < 0.001).

Lateral errors for each group and localization test are represented in Figure 2. No significant difference was found between the three groups in L_1_ (all *p* > 0.05). In L_2_, while no significant difference was found between ${\text{G}}_{10^{{}^{\circ}}}$ and G_C_ (*t* = −1.40, *p* = 0.17), a significant difference was found between ${\text{G}}_{20^{{}^{\circ}}}$ and G_C_ (*t* = −3.90, *p* < 0.001) and ${\text{G}}_{20^{{}^{\circ}}}$ and ${\text{G}}_{10^{{}^{\circ}}}$ (*t* = −2.50, *p* = 0.015). The difference between L_2_ and L_1_ was 5.58° leftward for ${\text{G}}_{20^{{}^{\circ}}}$ compared to G_C_ and 2.58° leftward for ${\text{G}}_{20^{{}^{\circ}}}$ compared to ${\text{G}}_{10^{{}^{\circ}}}$. The results of the MLM on lateral errors for each group are displayed in Table 3. While no significant difference between L_1_ and L_2_ was found for ${\text{G}}_{10^{{}^{\circ}}}$ (*t* = 0.92, *p* = 0.37) and ${\text{G}}_{20^{{}^{\circ}}}$ (*t* = −1.45, *p* = 0.16), a significant rightward deviation in L_2_ compared to L_1_ was found for G_C_ (*t* = 3.79, *p* = 0.001).

**Table 3:** Fixed effects for linear mixed models predicting lateral errors in each group (G_C_ = control group, ${\text{G}}_{10^{{}^{\circ}}}$ = group with 10° lateral deviation, ${\text{G}}_{20^{{}^{\circ}}}$ = group with 20° lateral deviation; ^*^p < 0.05, ^**^p < 0.01).

**Group**	**Estimate**	**Standard error**	**95% confidence interval**	***t***	***p***
**G**_C_					
Intercept	3.74	1.59	[0.63, 6.85]	2.36	0.029^*^
Localization test (L_2_-L_1_)	3.96	1.05	[1.91, 6.01]	3.79	0.001^**^
${\text{G}}_{10^{{}^{\circ}}}$					
Intercept	2.35	1.43	[−0.45, 5.14]	1.64	0.12
Localization test (L_2_-L_1_)	0.96	1.05	[−1.09, 3.02]	0.92	0.37
${\text{G}}_{20^{{}^{\circ}}}$					
Intercept	−1.55	1.06	[−3.64, 0.54]	−1.46	0.16
Localization test (L_2_-L_1_)	−1.62	1.12	[−3.80, 0.57]	−1.45	0.16

## 4 Discussion

The present study aimed at inducing an audio-proprioceptive misalignment in two experimental groups (${\text{G}}_{10^{{}^{\circ}}}$ and ${\text{G}}_{20^{{}^{\circ}}}$) thanks to a short session of active training in which the participant's hand was sonified at 10° or 20° to the right of its actual position. These two groups were compared to a control group in which no deviation was induced (G_C_). We hypothesized a more leftward sound localization for ${\text{G}}_{10^{{}^{\circ}}}$ and ${\text{G}}_{20^{{}^{\circ}}}$ compared to the G_C_ in the post-adaptation localization test, with a stronger effect for ${\text{G}}_{20^{{}^{\circ}}}$.

First, our three groups did not significantly differ in the number of targets found during the adaptation task, suggesting that the performance in this task did not seem to be affected by the lateral deviation induced for ${\text{G}}_{10^{{}^{\circ}}}$ and ${\text{G}}_{20^{{}^{\circ}}}$.

For the localization tasks, the three groups did not significantly differ in lateral errors, absolute polar errors, and front-back confusions in L_1_, suggesting that the three groups were quite equivalent in terms of sound localization abilities before the training. Only a significant difference between G_C_ and ${\text{G}}_{20^{{}^{\circ}}}$ was found for absolute lateral errors with smaller absolute lateral errors in ${\text{G}}_{20^{{}^{\circ}}}$ than in G_C_. After the training, in L_2_, the three groups still did not differ in polar errors and front-back confusions. Moreover, no improvement was found between localization tests for absolute lateral and polar errors, suggesting that the training, including the non-deviated one, did not improve the sound localization accuracy. Only a statistical trend for a decrease in front-back confusions between L_1_ and L_2_ can be highlighted. This agrees with previous studies showing that one single session is not sufficient to improve sound localization and suggests that more sessions are preconized to obtain a benefit (not found in all participants; Parseihian and Katz, 2012; Bouchara et al., 2019; Stitt et al., 2019).

As expected, the mixed linear model revealed a significant interaction between the factors Group and Localization test for lateral errors, suggesting an effect of the lateral deviation induced during training. The difference between L_2_ and L_1_ was 3.00° leftward for ${\text{G}}_{10^{{}^{\circ}}}$ compared to G_C_, but not significant_._ In contrast, the difference between L_2_ and L_1_ was significant and 5.58° leftward for ${\text{G}}_{20^{{}^{\circ}}}$ compared to G_C_. This latter result was in line with our hypothesis. However, counter-intuitively, no significant difference was shown between both localization tests for ${\text{G}}_{10^{{}^{\circ}}}$ and ${\text{G}}_{20^{{}^{\circ}}}$, while a leftward bias was expected. Instead, a significant rightward deviation was shown for G_C_, while no lateral shift was expected given that no bias was induced. The most plausible explanation must be that a fatigue effect skewed the overall results of the three groups to the right. Indeed, L_2_ was carried out at the end of the 30-min session involving frequent movements of the dominant arm (90° forward elevation), which may have generated a fatigue effect over trials and thus a progressive muscular relaxation, leading to a slight release of the arm with a natural deviation of the limb to the right for right-handers, and to the left for left-handers (53 of our 60 participants were right-handers). Previous studies carried out with similar tasks did not allow us to make any conclusions concerning this hypothesis as only absolute errors were analyzed (Parseihian and Katz, 2012; Bouchara et al., 2019; Stitt et al., 2019). If this effect is proven, it can be a huge limitation for extending this training to a brain-damaged population suffering from motor limitations, underlying the need to adapt the current training to implement it in clinical settings.

Additional limitations could also be highlighted. Although one session appears sufficient to show a significant effect between G_C_ and ${\text{G}}_{20^{{}^{\circ}}}$, proposing more sessions should be necessary to understand learning over time and dissociate it from other effects such as fatigue effect (Parseihian and Katz, 2012; Bouchara et al., 2019). Another limit could be the choice of the deviation amplitudes (10° and 20°). This choice was based on the literature to propose an experiment comparable to experiments using PA (Jacquin-Courtois et al., 2013; Bourgeois et al., 2021). However, localization performance is not equivalent between hearing and vision, with less precision for the auditory modality (Blauert, 1996). Although HRTF were selected for each participant in the present study, sound localization can be described as poor and very heterogeneous from one participant to another. It is worth mentioning that, when questioned at the end of the experiment, none of the participants of ${\text{G}}_{10^{{}^{\circ}}}$ and ${\text{G}}_{20^{{}^{\circ}}}$ mentioned having noticed the lateral shift. Therefore, as the auditory modality is less reliable than the visual one, one hypothesis could be that the amplitude of the deviation needs to be larger, which is consistent with the multisensory integration model (Ernst and Bülthoff, 2004). Future studies will, thus, need to test larger amplitudes of deviation to determine the best amplitude to propose for hearing.

Although other factors, such as fatigue, may have cumulated with the training after-effect, our study contributes to and offers perspectives for future studies aimed at a better understanding of auditory adaptation mechanisms, which is essential for improving the way hearing can be used in multisensory training, such as in rehabilitation programs of spatial cognition. The use of hearing could be crucial in some contexts, particularly in the case of visual impairments (Gori, 2015; Cappagli et al., 2019). A growing body of evidence also supports multisensory training, including the auditory modality, rather than unisensory training to improve USN (Frassinetti et al., 2002; Guilbert et al., 2014). Although PA was already proposed through immersive VR to healthy participants (Bourgeois et al., 2021; Cho et al., 2022) and patients with USN (Chen et al., 2022), none of these programs included spatial sounds, whereas immersive VR offers the advantage of easily implementing them. Although the coupling of hearing and vision in PA still needs to be studied, adding spatial hearing to PA programs could be a promising way to reinforce after-effects and optimize their benefits.

## Data availability statement

The datasets presented in this study can be found in online repositories. The names of the repository/repositories and accession number(s) can be found at: https://osf.io/hq8wp/.

## Ethics statement

The study involving humans was approved by Paris Cité University Ethical Committee (N°IRB: 00012019-24). The study was conducted in accordance with the local legislation and institutional requirements. The participants provided their written informed consent to participate in this study.

## Author contributions

AG: Conceptualization, Formal analysis, Funding acquisition, Investigation, Supervision, Writing – original draft. T-GB: Conceptualization, Investigation, Software, Writing – review & editing. TB: Conceptualization, Software, Supervision, Writing – review & editing.
